# Evolving landscape of potassium-competitive acid blockers: toward disease-specific use beyond acid suppression

**DOI:** 10.1007/s00535-026-02422-4

**Published:** 2026-05-19

**Authors:** Hideki Mori, Hidekazu Suzuki

**Affiliations:** 1 Division of Gastroenterology and Hepatology, Department of Internal Medicine, Keio University School of Medicine, 35 Shinanomachi, Shinjuku-ku, Tokyo, 160-8582 Japan; 2 Division of Gastroenterology and Hepatology, Department of Internal Medicine, Tokai University School of Medicine, 143 Shimokasuya, Isehara, Kanagawa 259-1193 Japan

**Keywords:** Potassium-competitive acid blockers, Proton pump inhibitors, Gastroesophageal reflux, *Helicobacter pylori*, Anti-ulcer agents

## Abstract

Potassium-competitive acid blockers (P-CABs) represent a major advancement in acid suppression by achieving rapid and sustained inhibition of gastric acid secretion. However, stronger acid suppression does not necessarily lead to better clinical outcomes. The clinical benefits of P-CABs vary according to the disease. In gastroesophageal reflux disease, P-CABs achieve healing rates for erosive esophagitis that are comparable to those of proton pump inhibitors (PPIs), with possible advantages in severe cases; however, consistent superiority in symptom control is lacking. The evidence for functional dyspepsia also remains insufficient. By contrast, in *Helicobacter pylori* eradication, P-CAB-based regimens have demonstrated clear advantages, particularly in the presence of antibiotic resistance. In peptic ulcer disease, the efficacy of P-CABs is comparable to that of PPIs. Long-term safety, including the effects of sustained hypergastrinemia, remains uncertain. Furthermore, symptom improvement and mucosal healing are not always concordant, thus highlighting the need to define therapeutic goals. Therefore, P-CABs should be used selectively on the basis of disease-specific requirements rather than pharmacological potency alone. An individualized approach is essential to optimize outcomes and avoid the unnecessary escalation of acid-suppressive therapy.

## Introduction

The potent suppression of gastric acid secretion is a cornerstone of the management of acid-related gastrointestinal disorders. For more than three decades, proton pump inhibitors (PPIs) have been the standard therapy for conditions, such as gastroesophageal reflux disease (GERD), peptic ulcer disease, and *Helicobacter pylori* infection [1–4]. However, several limitations of PPIs, including delayed onset of action, incomplete nocturnal acid suppression, and interindividual variability related to CYP2C19 polymorphisms, have prompted the development of alternative acid-suppressive agents [5].

Potassium-competitive acid blockers (P-CABs) have emerged as a new class of acid inhibitors targeting the gastric H⁺/K⁺-ATPase. Vonoprazan was first introduced in Japan in 2015, and its widespread use over the past decade has been accompanied by the development of other P-CAB agents in several regions. Consequently, clinical evidence has accumulated regarding their therapeutic roles in acid-related diseases.

Although P-CABs are recognized as potent acid suppressants, stronger pharmacological inhibition of gastric acid secretion does not necessarily translate into better clinical outcomes. The therapeutic benefit of acid suppression depends not only on the potency of the drug but also on the underlying disease mechanisms and the clinical goals of treatment. For example, in GERD, mucosal healing of erosive esophagitis and improvement of reflux symptoms are not always concordant [6]. Similarly, in peptic ulcer disease, the therapeutic considerations differ between spontaneous and procedure-related artificial ulcers following endoscopic intervention. In clinical practice, the prevention of delayed bleeding is a key concern, particularly in post-procedural ulcers; from a pathophysiological perspective, ulcer healing is commonly used as a surrogate endpoint for treatment efficacy [7]. However, the clinical relevance of ulcer healing as a surrogate marker remains uncertain because it may not necessarily translate into a reduced risk of clinically meaningful outcomes such as delayed bleeding [7].

By contrast, in *H. pylori* eradication therapy, treatment success is typically defined as a binary outcome (i.e., or failure) rather than symptom improvement or mucosal healing. In this context, treatment efficacy is strongly influenced by antibiotic resistance patterns in addition to intragastric pH control [4, 8–10]. Considering that reinfection after successful eradication is relatively uncommon, achieving a high eradication rate is the primary therapeutic goal [11].

These observations suggest that the clinical positioning of potent acid suppressants should be considered in the context of disease-specific therapeutic requirements rather than pharmacological potency alone. In this review, we summarize the pharmacological characteristics of P-CABs and discuss their clinical roles in major acid-related gastrointestinal diseases, including GERD, peptic ulcer disease, and *H. pylori* infection, with a particular emphasis on the disease-specific requirements for acid suppression and their implications for clinical practice.

## Pharmacological characteristics of P-CABs

P-CABs inhibit gastric acid secretion via a mechanism distinct from that of PPIs. Although PPIs irreversibly inhibit the H⁺/K⁺-ATPase after acid-dependent activation within parietal cells, P-CABs directly and reversibly block the potassium-binding site of the proton pump [12].

This pharmacological difference accounts for the characteristic features of P-CABs. First, P-CABs demonstrate a rapid onset of action because they do not require acid-mediated activation [5]. Second, they achieve more potent and sustained intragastric pH control, including during nighttime, when PPIs may show incomplete acid suppression [5].

Metabolic pathways also differ among these drug classes. Most PPIs are primarily metabolized by CYP2C19, thus leading to considerable interindividual variability in acid suppression. By contrast, vonoprazan and several other P-CABs are predominantly metabolized via CYP3A4, thus resulting in more stable pharmacokinetics across patients [13, 14].

The influence of CYP2C19 polymorphisms also differs across populations, with Asian populations exhibiting a higher prevalence of poor metabolizers than Western populations [15]. This may contribute to the more pronounced variability in PPI efficacy in Western populations, while the pharmacokinetic stability of P-CABs is less affected by genetic differences. Most clinical evidence for P-CABs has been generated in Asian populations; therefore, the generalizability of these findings to other populations requires further investigation.

Owing to these pharmacological characteristics, P-CABs can provide stronger and more consistent acid suppression than conventional PPIs. However, the clinical significance of this advantage depends on the disease-specific requirements for acid suppression. The level and duration of intragastric pH control required to achieve therapeutic benefits vary across diseases, as reflected by the differences in pH holding-time targets. For example, an intragastric pH threshold ≥ 4 is generally considered sufficient for mucosal healing in GERD, whereas a higher pH (≥ 5) is required to promote effective *H. pylori* eradication [16, 17]. Therefore, whether more potent acid suppression translates to improved outcomes should be interpreted in the context of the underlying pathophysiology and treatment goals.

## Disease-specific clinical positioning of P-CABs

### GERD

The therapeutic goals of GERD management can be broadly categorized into two domains: mucosal healing of erosive esophagitis and symptom control, including in non-erosive reflux disease (NERD). The clinical relevance of potent acid suppression may differ between these domains.

#### Healing of erosive esophagitis

The healing rates of erosive esophagitis achieved with P-CABs were comparable to those obtained with PPIs. However, across clinical trials, the consistent superiority of P-CABs over PPIs has not been uniformly demonstrated in unselected patient populations. The AGA Clinical Practice Update also suggests that P-CABs should not be routinely used as first-line therapy in patients with mild erosive esophagitis (LA grade A/B), whereas they may be considered in patients with more severe disease (LA grade C/D) [18]. This perspective is consistent with clinical evidence indicating that the benefits of P-CABs may be more apparent in specific clinical settings. In particular, potential advantages have been reported in severe erosive esophagitis, PPI-refractory disease, earlier mucosal healing (especially at 4 weeks), and maintenance therapy with reduced relapse rates, particularly with vonoprazan.

Among the currently available P-CABs, vonoprazan has the most extensive clinical evidence of efficacy (Table 1A). Phase III trials in Japan and the United States have demonstrated the non-inferiority of vonoprazan to PPIs in terms of healing and maintenance of remission, with signals suggesting greater efficacy in patients with severe erosive esophagitis and in PPI-refractory cases [19–21]. In patients with severe erosive esophagitis (LA grade C/D), vonoprazan demonstrated higher healing rates than PPIs (88.0% vs. 63.9% at week 2 and 96.0% vs. 80.6% at week 4), thus indicating a clinically meaningful advantage in this subgroup [19]. In maintenance therapy, randomized trials have shown the non-inferiority of vonoprazan to lansoprazole, with post-hoc and subsequent Asian studies suggesting lower relapse rates and potential superiority; however, the primary study designs were based on non-inferiority [22, 23].

**Table 1:** Efficacy of P-CABs versus proton pump inhibitors in GERD: erosive esophagitis and symptom control

Study	Country	P-CAB	Comparator	Population	Endpoint	Duration	Key result	Interpretation
*A: Endoscopic healing of erosive esophagitis*								
Ashida, et al. [18]	Japan	Vonoprazan 20 mg	Lansoprazole 30 mg	EE (LA A–D)	Healing	8-week	Non-inferior overall; higher efficacy in severe cases	Benefit in severe EE
Laine, et al. [19]	US/Europe	Vonoprazan 20 mg	Lansoprazole 30 mg	EE (LA A–D)	Healing/maintenance	8-week	Non-inferior; superiority in severe cases	Benefit in severe EE
Lee, et al. [23]	Korea	Tegoprazan 50 mg	Esomeprazole 40 mg	EE (LA A–D)	Healing	8-week	Non-inferior; similar healing rates between groups	Non-inferior to PPI; no clear superiority signal
Zhu, et al. [24]	China	Tegoprazan 50 mg	Esomeprazole 40 mg	EE (LA A–D)	Healing	8-week	Non-inferior; similar healing rates between groups	Non-inferior to PPI; no clear superiority signal
Lee, et al. [25]	Korea	Fexuprazan 40 mg	Esomeprazole 40 mg	EE (LA A–D)	Healing	8-week	Non-inferior; similar healing rates between groups	Non-inferior to PPI; no clear superiority signal
Oh, et al. [27]	Korea	Zastaprazan 20 mg	Esomeprazole 40 mg	EE (LA A–D)	Healing	8-week	Non-inferior overall; numerically higher healing rates in severe cases	Non-inferior overall; trend toward improved efficacy in severe cases
Sharma, et al. [28]	US/Europe	Linaprazan glurate 50–200 mg	Lansoprazole 30 mg	EE (LA C/D or PPI partial responders)	Healing	4-week	Higher healing rates vs lansoprazole	Possible efficacy advantage, but inconclusive due to inconsistent dose–response
Chen, et al. [29]	China	Keverprazan 20 mg	Lansoprazole 30 mg	EE (LA A–D)	Healing	8-week	Non-inferior overall; numerically higher healing rates vs lansoprazole	Non-inferior to PPI; modest numerical advantage without confirmed superiority
*B: Symptom control*								
Laine et al. [19]	US/Europe	Vonoprazan 20 mg	Lansoprazole 30 mg	EE (LA A–D)	24-h heartburn-free days	Up to 8 weeks	Non-inferior in heartburn-free days, with no superiority in overall symptom control	Comparable symptom control to PPI overall
Zhu et al. [24]	China	Tegoprazan 50 mg	Esomeprazole 40 mg	EE (LA A–D)	Symptom improvement; QoL	4–8 weeks	Non-inferior symptom improvement	Non-inferior to PPI; no clear superiority signal
Oh et al. [27]	Korea	Zastaprazan 20 mg	Esomeprazole 40 mg	EE (LA A–D)	Symptom response; GERD-HRQL	Up to 8 weeks	Non-inferior symptom improvement	Non-inferior to PPI; no clear superiority signal
Tack et al. [30]	Europe	Vonoprazan 20/40 mg	Esomeprazole 40 mg	Symptomatic GERD	Heartburn-free 24-h periods	4 weeks	No difference in symptom control between groups	Comparable symptom control in partial PPI responders
Oshima et al. [32]	Japan	Vonoprazan 20 mg	Lansoprazole 30 mg	EE with heartburn	Time to relief; nocturnal relief	Up to 2 weeks	Faster heartburn relief; better nocturnal relief	Possible early symptom-relief advantage vs PPI
Kang et al. [33]	Korea	Tegoprazan 50 mg (on-demand)	Esomeprazole 20 mg (on-demand)	GERD (NERD or ERD)	Patient satisfaction; time to symptom improvement	8 weeks	Similar overall symptom control, with faster symptom relief vs PPI	Comparable overall control, with faster on-demand relief
Kim et al. [34]	Korea	Tegoprazan 50 mg	Esomeprazole 40 mg	GERD with nighttime heartburn/sleep disturbance	Nighttime heartburn relief; sleep quality	2 weeks	No significant difference in primary endpoint; numerically faster nocturnal symptom relief	Possible advantage for nocturnal symptoms, but evidence remains limited
Oh et al. [35]	Korea	Fexuprazan 40 mg	Esomeprazole 40 mg	GERD with nocturnal reflux symptoms	Change in nocturnal symptom severity	4 weeks	Greater improvement in nocturnal symptoms vs PPI	Superiority signal in nocturnal GERD
Kwon et al. [37]	Korea	Tegoprazan 50 mg alternate-day	Lansoprazole 15 mg daily	NERD, symptomatic maintenance therapy	Proportion of symptom-free days	8 weeks	No difference in symptom-free days or symptom scores between groups	Comparable maintenance symptom control

*EE* erosive esophagitis, *GERD* gastroesophageal reflux disease, *NERD* non-erosive reflux disease, *ERD* erosive reflux disease, *QoL* quality of life, *GERD-HRQL* GERD health-related quality of life, *LA* Los Angeles classification

Tegoprazan has been evaluated in phase III randomized trials in Korea and China, where it demonstrated non-inferiority to esomeprazole in the 8-week healing rate of erosive esophagitis [24, 25]. Unlike vonoprazan, clear evidence demonstrating the superior efficacy of tegoprazan in patients with severe erosive esophagitis has not yet been established, and the overall efficacy appears to be comparable to that of PPIs.

Similarly, phase III trials in Korea and China have shown that fexuprazan is non-inferior to esomeprazole for the healing of erosive esophagitis [26, 27]. Owing to the small number of severe cases, its efficacy in this subgroup has not been clearly demonstrated. On the basis of currently available data, fexuprazan appears to be effective for the treatment of erosive esophagitis, although its superiority over PPIs has not been consistently demonstrated.

Zastaprazan exhibited distinct findings. Although its 8-week cumulative healing rate was non-inferior to esomeprazole, its 4-week healing rate was significantly higher in the full analysis set [28]. In severe cases (LA grade C/D), higher healing rates were observed with Zastaprazan (100% vs. 66.7% at week 4). However, the difference was not statistically significant owing to the small sample size. Therefore, the apparent advantages at earlier time points should be interpreted with caution [28].

A recent dose-finding study showed that linaprazan glurate had higher 4-week healing rates than lansoprazole when all doses were pooled [29]. However, the dose–response relationship was not entirely consistent, thus suggesting that further studies are required to clarify the optimal dosing strategy.

Keverprazan also demonstrated non-inferiority to lansoprazole in a phase III randomized trial conducted in China. Similar to other P-CABs, the available data support the efficacy of keverprazan as an alternative to PPIs; however, clear superiority has not yet been established [30]. In addition, its efficacy in patients with severe erosive esophagitis has not been clearly demonstrated. In a subgroup analysis, the 8-week healing rates of keverprazan were similar between groups (66.7% vs. 60.9%) without a statistically significant difference, thus suggesting that any potential advantage may be limited.

#### Symptom control in GERD and NERD

Overall, the available clinical evidence suggests that P-CABs provide symptom control in GERD that is broadly comparable to that achieved with PPIs rather than consistently demonstrating clear superiority (Table 1B). Several randomized trials evaluating vonoprazan and other P-CABs have shown similar improvements in patient-reported outcomes, including heartburn-free days; heartburn-free 24-h periods; and changes in GERD symptom scores, such as the Frequency Scale for the Symptoms of GERD [20, 25, 28, 31, 32]. In many of these studies, both P-CABs and PPIs produced significant symptom improvements over time, whereas the between-group differences were generally modest or not statistically significant. Consistent with these findings, the AGA Clinical Practice Update suggests that P-CABs should not be routinely used as first-line therapy in patients with uninvestigated heartburn or non-erosive reflux disease, and that evidence remains insufficient to support their use in on-demand therapy [18].

However, their rapid and sustained acid suppression raises the possibility that P-CABs may offer clinical advantages in selected situations. Some studies have suggested the potential advantages of P-CABs for early symptom relief, which may be due to rapid and sustained acid suppression [33, 34]. For example, vonoprazan has been reported to produce earlier relief from heartburn during the first week of treatment, although the overall symptom scores after several weeks were comparable to those observed with PPIs [33]. Similarly, several studies on vonoprazan, tegoprazan and fexuprazan have focused on nocturnal symptoms, a clinical area in which incomplete acid suppression with PPIs has been recognized [33, 35, 36]. These studies suggest that P-CABs may provide more effective control of nighttime symptoms. However, evidence remains limited, and findings have been inconsistent across trials.

Another area of interest is the potential use of P-CABs in on-demand therapies for GERD and non-erosive reflux disease. Given that P-CABs do not require acid activation and exert rapid anti-secretory effects, they may offer rapid symptom relief when administered intermittently. Recent studies from Korea and Japan have explored on-demand or intermittent dosing strategies and reported prompt symptom improvement after dosing; therefore, P-CABs may be well suited for such treatment approaches [34, 37–39]. However, further comparative studies are required to clarify whether these pharmacological advantages translate into clinically meaningful improvements over PPIs in routine symptom management.

### *H. pylori* eradication

Considering that *H. pylori* antimicrobial resistance profiles differ not only across continents, but also within individual countries and regions, the choice of first-line eradication regimen should be interpreted in a regional context. In particular, resistance to clarithromycin and metronidazole has a major influence on the selection and expected efficacy of first-line therapy. The AGA Clinical Practice Update recommends the use of P-CABs in place of PPIs for most patients undergoing *H. pylori* eradication therapy [18]. However, the magnitude of this benefit may vary depending on the specific P-CAB and antimicrobial resistance patterns, as discussed in the following sections.

#### Japan

Japan has a distinctive antimicrobial resistance profile for *H. pylori*, which is characterized by relatively high clarithromycin resistance (approximately 40%) and very low metronidazole resistance (generally < 5%), thus influencing the choice of eradication regimens [4, 40]. However, because of insurance coverage policies, standard triple therapy remains the approved first-line regimen in Japan [41].

In a pivotal Japanese phase III, randomized, double-blind trial, vonoprazan-based triple therapy achieved significantly higher first-line eradication rates than lansoprazole-based triple therapy (92.6% vs. 75.9% in intention-to-treat analysis), with a particularly substantial advantage in patients with clarithromycin-resistant strains [42] (Table 2). In the same study, second-line vonoprazan–amoxicillin–metronidazole therapy demonstrated excellent efficacy by achieving an eradication rate of approximately 98% [42]. Subsequent Japanese studies further supported the effectiveness of vonoprazan-based first-line regimens [43–45]. In particular, a randomized study by Suzuki et al. demonstrated that vonoprazan–amoxicillin dual therapy was non-inferior to vonoprazan–amoxicillin–clarithromycin triple therapy [46]. This finding provided an important basis for the subsequent development of P-CAB-amoxicillin dual therapy strategies, which have been widely investigated, particularly in China.

**Table 2:** Efficacy of P-CAB–based eradication regimens for *Helicobacter pylori* outside China

Region	Study	Regimen	Duration	Comparator	Duration	Line	Key result	Interpretation
Japan	Murakami et al.[40]	VPZ 20 mg b.i.d., AMX 750 mg b.i.d., CLR 200/400 mg b.i.d	7	LPZ 30 mg b.i.d., AMX 750 mg b.i.d., CLR 200/400 mg b.i.d	7	First	92.6% vs 75.9%	VAC was superior to LAC (especially in CLR-resistant strains)
Japan	Murakami et al.[40]	VPZ 20 mg b.i.d., AMX 750 mg b.i.d., MTZ 250 mg b.i.d	7	–		Second	98.0%	Highly effective
Japan	Suzuki et al.[44]	VPZ 20 mg b.i.d., AMX 750 mg b.i.d	7	VPZ 20 mg b.i.d., AMX 750 mg b.i.d., CLR 200/400 mg b.i.d	7	First	84.5% vs 89.2%	VA-dual was non-inferior to VAC-triple
Japan	Sue et al.[45]	VPZ 20 mg b.i.d., AMX 750 mg b.i.d., STFX 100 mg b.i.d	7	PPI b.i.d., AMX 750 mg b.i.d., STFX 100 mg b.i.d	7	Third	75.8% vs 53.3%	VAS was superior to PAS
Korea	Park et al.[56]	TPZ 50 mg b.i.d., AMX 1000 mg b.i.d., CLR 500 mg b.i.d	14	LPZ 30 mg b.i.d., AMX 1000 mg b.i.d., CLR 500 mg b.i.d	14	First	86.0%, 85.5% vs 78.7%	Both TAC regimens were non-inferior to LAC
		TPZ 100 mg b.i.d., AMX 1000 mg b.i.d., CLR 500 mg b.i.d	14					
Korea	Choi et al.[58]	TPZ 50 mg b.i.d., AMX 1000 mg b.i.d., CLR 500 mg b.i.d	7	LPZ 30 mg b.i.d., AMX 1000 mg b.i.d., CLR 500 mg b.i.d	7	First	62.9% vs 60.6%	TAC was non-inferior to LAC
Korea	Kim et al.[59]	TPZ 50 mg b.i.d., TET 500 mg q.i.d., MTZ 500 mg t.i.d., bismuth 300 mg q.i.d	14	LPZ 30 mg b.i.d., TET 500 mg q.i.d., MTZ 500 mg t.i.d., bismuth 300 mg q.i.d	14	First	80.0% vs 77.4%	TBMT was non-inferior to LBMT
Taiwan	Chiu et al.[79]	VPZ 20 mg b.i.d., AMX 1000 mg b.i.d., CLR 500 mg b.i.d	7	①LPZ 30 mg b.i.d., AMX 1000 mg b.i.d., ②LPZ 30 mg b.i.d., CLR 500 mg b.i.d., MTZ 500 mg b.i.d	14 (7 + 7)	First	88.6% vs 81.8%	7-day VAC was non-inferior to 14-day Sequential
Thailand	Bunchorntavakul et al.[82]	VPZ 20 mg b.i.d., AMX 1000 mg b.i.d., CLR 500 mg b.i.d	7	OPZ 20 mg b.i.d., AMX 1000 mg b.i.d., CLR 500 mg b.i.d	14	First	98.3% vs 93.1%	Comparable efficacy
Singapore	Ang et al.[83]	VPZ 20 mg b.i.d., AMX 1000 mg b.i.d., CLR 500 mg b.i.d	7	PPI b.i.d., AMX 1000 mg b.i.d., CLR 500 mg b.i.d	14	First	87.4% vs 88.0%	7-day TAC was non-inferior to 14-day PAC
US/Europe	Chey et al.[84]	VPZ 20 mg b.i.d., AMX 1000 mg t.i.d	14	LPZ 30 mg b.i.d., AMX 1000 mg b.i.d., CLR 500 mg b.i.d	14	First	84.7% (VA) 78.5% (VAC), vs 78.8% (LAC)	VA/VAC were non-inferior to LAC, but superior in CLR-resistant strains
		VPZ 20 mg b.i.d., AMX 1000 mg b.i.d., CLR 500 mg b.i.d	14					
Egypt	Lashen et al.[85]	VPZ 20 mg b.i.d., AMX b.i.d., CLR b.i.d	14	EPZ 20 mg b.i.d., AMX b.i.d., CLR b.i.d	14	First	87.6% vs 76.9%	VAC was superior to EAC
Pakistan	Waqar et al.[87]	VPZ 20 mg b.i.d., AMX 1000 mg b.i.d., LVFX 500 mg q.d	7	EPZ 20 mg b.i.d., AMX 1000 mg b.i.d., LVFX 500 mg q.d	14	First	95.0% vs 93.4%	Comparable efficacy

*VA* vonoprazan + amoxicillin, *VAC* vonoprazan + amoxicillin + clarithromycin, *VAM* vonoprazan + amoxicillin + metronidazole, *VAS* vonoprazan + amoxicillin + sitafloxacin, *VAL* vonoprazan + amoxicillin + levofloxacin, *TAC* tegoprazan + amoxicillin + clarithromycin, *TBMT* tegoprazan + bismuth + metronidazole + tetracycline, *LAC* lansoprazole + amoxicillin + clarithromycin, *PAS* PPI + amoxicillin + sitafloxacin, *PAC* PPI + amoxicillin + clarithromycin, *LBMT* lansoprazole + bismuth + metronidazole + tetracycline, *EAC* esomeprazole + amoxicillin + clarithromycin, *AMX* amoxicillin, *CLR* clarithromycin, *MTZ* metronidazole, *STFX*,sitafloxacin, *LVFX* levofloxacin, *TET* tetracycline, *PPI* proton pump inhibitor, *b.i.d.* twice daily, *t.i.d.* three times daily, *q.i.d.* four times daily; q.d., once daily

A randomized trial demonstrated that vonoprazan–amoxicillin–sitafloxacin was superior to esomeprazole-based therapy for third-line eradication [47]. In addition, accumulating real-world data suggest favorable outcomes with vonoprazan-based sitafloxacin- or rifabutin-containing regimens for third-line and later eradication therapies [48–55].

It should be noted that most eradication regimens in Japan are administered for 7 days, which differ from the longer treatment durations commonly used in other countries.

#### Korea

A nationwide, multicenter surveillance study in Korea reported *H. pylori* antimicrobial resistance rates of approximately 17.8% and 29.5% for clarithromycin and metronidazole, respectively. Therefore, both clarithromycin and metronidazole resistance should be considered when selecting empirical eradication therapy [56]. Furthermore, most of the available evidence in Korea for P-CAB-based *H. pylori* eradication was generated using tegoprazan (Table 2). The main body of evidence consists of comparisons between tegoprazan- and lansoprazole-based clarithromycin triple therapies [57–60]. Overall, tegoprazan-based triple therapy has shown non-inferior eradication efficacy compared with lansoprazole-based therapy, with a tendency toward better outcomes at the 100-mg per dose than at the 50-mg per dose [58]. However, the advantages of tegoprazan-based triple therapy in patients with clarithromycin-resistant strains remain limited and have not been consistently demonstrated. Evidence for tegoprazan-based bismuth quadruple or sequential therapy is also available, but these regimens have generally shown efficacy that is comparable to that of PPI-based therapy rather than clear superiority [61, 62].

#### China

The prevalence of *H. pylori* antimicrobial resistance in China shows substantial geographical variability. Clarithromycin resistance has been reported to range from approximately 20% to over 40% (even exceeding 50% in some regions), whereas metronidazole resistance is consistently high nationwide and often exceeds 60–70% [63]. In addition, multidrug resistance, particularly to clarithromycin and metronidazole, has been frequently observed. These resistance patterns have historically led to the widespread adoption of clarithromycin-free regimens, such as bismuth quadruple and concomitant therapies. More recently, with the introduction of P-CABs, treatment strategies have shifted toward simplified regimens, particularly vonoprazan- or tegoprazan-based amoxicillin dual therapy, which has been extensively investigated in China (Table 3).

**Table 3:** P-CAB-based eradication strategies in China: a concept-based summary EPZ, esomeprazole; KPZ, keverprazan; RPZ, rabeprazole; TPZ, tegoprazan; VPZ, vonoprazan; AMX, amoxicillin; CLR,clarithromycin; MTZ, metronidazole; TET, tetracycline

Study	Key comparison	P-CAB	Regimen	Duration	Comparator	Duration	Line	Key finding	Interpretation
*A. Dual therapy vs standard regimens*									
Zhang et al.[62]	VA vs VAC, V-BQT	Vonoprazan	VPZ 20 mg b.i.d., AMX 1000 mg t.i.d	10	VPZ 20 mg b.i.d., AMX 1000 mg b.i.d., CLR 500 mg b.i.d	10	First	92.0% vs 89.6%	VA-dual was non-inferior to V-BQT
					VPZ 20 mg b.i.d., AMX 1000 mg b.i.d., CLR 500 mg b.i.d., bismuth 240 mg b.i.d	10	First	92.0% vs 88.8%	
Cheung et al.[64]	VA vs VAC, E-BQT	Vonoprazan	VPZ 20 mg b.i.d., AMX 1000 mg t.i.d	14	VPZ 20 mg b.i.d., AMX 1000 mg b.i.d., CLR 500 mg b.i.d	14	First	96.0% vs 95.9%	Comparable
					EPZ 20 mg b.i.d., TET 500 mg t.i.d., MTZ 500 mg q.i.d., bismuth 220 mg q.i.d	14	First	96.0% vs 92.0%	VA-dual was non-inferior to BQT
Lin et al.[67]	TA vs E-BQT	Tegoprazan	TPZ 50 mg q.d., AMX 1000 mg t.i.d	14	EPZ 20 mg b.i.d., AMX 1000 mg b.i.d., CLR 500 mg b.i.d., bismuth 240 mg b.i.d	14	First	86.0% vs 85.1%	Both TA-dual was non-inferior to E-BQT
			TPZ 50 mg b.i.d., AMX 1000 mg t.i.d	14			First	86.0% vs 85.1%	
Yang et al.[68]	TA vs E-BQT	Tegoprazan	TPZ 50 mg b.i.d., AMX 750 mg q.i.d	14	EPZ 20 mg b.i.d., AMX 1000 mg b.i.d., MTZ 400 mg q.i.d., bismuth 600 mg b.i.d	14	First	68.1% vs 81.3%	TA did not achieve non-inferiority to E-BQT
Fan et al.[69]	TA vs E-BQT	Tegoprazan	TPZ 50 mg b.i.d., AMX 1000 mg t.i.d	10	EPZ 20 mg b.i.d., AMX 1000 mg b.i.d.,TET 500 mg t.i.d., bismuth 220 mg q.i.d	14	First	73.7% vs 75.0%	14-day TA was non-inferior to E-BQT
			TPZ 50 mg b.i.d., AMX 1000 mg t.i.d	14				53.9% vs 75.0%	10-day TA did not achieve non-inferiority to E-BQT
Wei et al.[70]	KA vs E-BQT	Keverprazan	KPZ 20 mg b.i.d., AMX 1000 mg t.i.d	14	EPZ 20 mg b.i.d., AMX 1000 mg b.i.d., CLR 500 mg b.i.d., bismuth 220 mg b.i.d	14	First	87.9% vs 84.2%	KA was non-inferior to E-BQT
B. Duration									
Song et al.[71]	10 vs 14 days	Vonoprazan	VPZ 20 mg b.i.d., AMX 1000 mg t.i.d	10	VPZ 20 mg b.i.d., AMX 1000 mg t.i.d	14	First	83.3% vs 88.0%	10-day VA did not achieve non-inferiority to 14-day VA
Lin et al.[72]	10 vs 14 days	Vonoprazan	VPZ 20 mg b.i.d., AMX 1000 mg t.i.d	10	VPZ 20 mg b.i.d., AMX 1000 mg t.i.d	14	First	89.6% vs 91.2%	10-day VA was non-inferior to 14-day VA
Peng et al.[73]	10 vs 14 days	Vonoprazan	VPZ 20 mg b.i.d., AMX 1000 mg b.i.d	10	VPZ 20 mg b.i.d., AMX 1000 mg b.i.d	14	First	79.7% vs 89.5%	10-day VA did not achieve non-inferiority to 14-day VA
Peng et al.[73]	10 vs 14 days	Vonoprazan	VPZ 20 mg b.i.d., AMX 750 mg q.i.d	10	VPZ 20 mg b.i.d., AMX 750 mg q.i.d	14	First	86.6% vs 89.5%	10-day VA was non-inferior to 14-day VA
*C. Amoxicillin dose*									
Wu et al.[74]	500 mg q.i.d. vs, 750 mg q.i.d., 1000 mg t.i.d	Vonoprazan	VPZ 20 mg b.i.d., AMX 500 mg q.i.d	14	VPZ 20 mg b.i.d., AMX 750 mg q.i.d	14	First	93.3% vs 93.6%	500 mg q.i.d. was sufficient
					VPZ 20 mg b.i.d., AMX 1000 mg t.i.d	14	First	93.3% vs 92.4%	
Hu et al.[75]	1000 mg b.i.d. vs 1000 mg t.i.d	Vonoprazan	VPZ 20 mg b.i.d., AMX 1000 mg b.i.d	14	VPZ 20 mg b.i.d., AMX 1000 mg t.i.d	14	First	85.3% vs 86.5%	1000 mg b.i.d. was sufficient
*D. Drug differences*									
Han et al.[77]	10-day VA vs 14-day RA	Vonoprazan	VPZ 20 mg b.i.d., AMX 1000 mg t.i.d	10	RPZ 10 mg t.i.d., AMX 1000 mg t.i.d	14	First	89.3% vs 84.9%	10-day VA was non-inferior to 14-day RA
Kong et al.[78]	TA vs EA	Tegoprazan	TPZ 50 mg b.i.d., AMX 750 mg q.i.d	14	EPZ 20 mg q.i.d., AMX 750 mg q.i.d	14	First	84.2% vs 85.8%	TA was non-inferior to EA

*VA* vonoprazan + amoxicillin, *VAC* vonoprazan + amoxicillin + clarithromycin, *V-BQT* vonoprazan + bismuth + quadruple therapy (amoxicillin + clarithromycin + bismuth), *TA* tegoprazan + amoxicillin, *KA* keverprazan + amoxicillin, *E-BQT* esomeprazole + bismuth + quadruple therapy, *EA* esomeprazole + amoxicillin, *RA* rabeprazole + amoxicillin

Evidence for P-CAB-amoxicillin dual therapy in China is best interpreted according to four clinical questions: comparison with other eradication regimens, optimal treatment duration, optimal amoxicillin dose, and the relative efficacy of P-CAB versus PPI-based dual therapy (Table 3A).

Overall, multiple randomized trials from China suggest that vonoprazan-based amoxicillin dual therapy can achieve eradication rates that are comparable to those of bismuth-containing quadruple regimens or clarithromycin-containing triple regimens, with the advantages of a simpler regimen, fewer adverse events, and lower cost [64–68].

By contrast, evidence for tegoprazan-based dual therapy remains controversial. Although some studies reported comparable efficacy to standard regimens [69], a recent randomized trial demonstrated that 14-day tegoprazan–amoxicillin dual therapy failed to achieve non-inferiority compared with bismuth quadruple therapy [70]. Another study reported that 14-day dual therapy was non-inferior to bismuth quadruple therapy, whereas 10-day therapy was considered unacceptable [71]. Data are limited for keverprazan, which has shown non-inferior efficacy to bismuth quadruple therapy in dual therapy regimens [72].

With regard to treatment duration, 14-day dual therapy provides more reliable eradication rates than shorter courses (Table 3B). For vonoprazan-based dual therapy, although some studies reported comparable efficacy between the 10- and 14-day regimens, others failed to demonstrate equivalence, thus suggesting that shorter durations cannot be considered a universal substitute for 14-day therapy [73–75].

Recent studies in China suggest that very high doses are not always necessary for vonoprazan-based amoxicillin dual therapy (Table 3C). Several studies suggest that high-dose amoxicillin (e.g., 3 g/day) may not be necessary in vonoprazan-based dual therapy and that a daily dose of approximately 2 g is sufficient to maintain efficacy [76, 77]. However, a 10-day regimen with 1 g amoxicillin twice daily showed suboptimal eradication rates in a single randomized study, thus suggesting that dosing frequency, rather than the total daily dose, may influence treatment efficacy [75, 78].

Finally, P-CAB-based dual therapy appears more robust than PPI-based dual therapy (Table 3D). In a randomized study, 10-day vonoprazan–amoxicillin therapy achieved higher eradication rates than 14-day rabeprazole-amoxicillin therapy, thus supporting the concept that stronger acid suppression enhances the efficacy of amoxicillin dual therapy [79]. By contrast, a separate randomized trial using tegoprazan showed that 14-day tegoprazan–amoxicillin was comparable with esomeprazole–amoxicillin, thus indicating that this effect may not be uniform across all P-CABs [80].

Available Chinese data suggest that vonoprazan-based amoxicillin dual therapy is a practical and increasingly evidence-based first-line treatment option. In contrast, the efficacy of tegoprazan-based dual therapy remains controversial, and data on keverprazan are limited. Amoxicillin doses of at least 2 g/day administered in divided doses appear to be generally sufficient, although the optimal regimen may vary across regions and study populations.

#### Other Asian countries and regions

However, evidence regarding vonoprazan-based eradication therapies in other Asian countries is limited (Table 2). In Taiwan, a study comparing vonoprazan-based triple therapy with extended sequential therapy demonstrated non-inferior eradication rates, with a higher efficacy observed in the vonoprazan group (88.6% vs. 81.8%) [81]. These findings support the potential advantages of vonoprazan-based standard triple regimens as simplified treatment options.

In Thailand, several randomized controlled trials have evaluated vonoprazan-containing regimens. Although acceptable eradication rates have been reported with 7-day vonoprazan-based standard triple therapy, the available studies are heterogeneous in terms of dosing regimens, treatment durations, and comparator therapies. Therefore, definitive conclusions remain difficult to obtain [82–84].

In Singapore, limited data suggest that vonoprazan-based regimens achieve eradication rates that are comparable to those of PPI-based therapy despite a shorter treatment duration (7 vs. 14 days); this finding supports the potential advantage of vonoprazan [85].

Evidence from these countries remains relatively sparse; however, vonoprazan-based standard triple therapy appears to provide consistent and acceptable eradication rates in different settings. Further studies are required to elucidate the role of P-CAB regimens in various environments with antimicrobial resistance.

#### Western countries

Evidence remains limited regarding the efficacy of vonoprazan-based eradication therapy in Western populations (Table 2). A large, randomized, phase III trial conducted in the United States and Europe demonstrated that vonoprazan-based triple therapy achieved eradication rates comparable to those of PPI-based triple therapy and showed clear superiority in patients with clarithromycin-resistant strains [86]. Vonoprazan–amoxicillin dual therapy also demonstrated non-inferiority to vonoprazan-based triple therapy and superiority to PPI-based triple therapy in clarithromycin-resistant infections. However, the interpretation of these findings is complicated by the differences in amoxicillin dosing regimens between the treatment arms, and the relative contribution of dual versus triple therapy remains uncertain. Overall, these results support the robustness of vonoprazan-based regimens, particularly in the setting of clarithromycin resistance; however, the optimal regimen selection requires further investigation.

#### Other regions

Evidence regarding vonoprazan-based eradication therapy in regions outside East Asia remains limited (Table 2). In Egypt, randomized studies comparing vonoprazan-based regimens with esomeprazole-based therapy reported higher eradication rates with vonoprazan-containing regimens [87, 88].

In Pakistan, several studies evaluated vonoprazan-based therapies for *H. pylori* eradication and suggested that vonoprazan-containing regimens may achieve acceptable eradication rates. However, the available evidence remains limited and heterogeneous [89, 90].

Further studies from diverse geographical regions are required to clarify the role of P-CAB-based regimens in different antimicrobial resistance settings.

### Peptic ulcer disease

Peptic ulcer disease should be considered separately according to the clinical context because the pathophysiology, therapeutic goals, and outcome measures differ among post-endoscopic artificial ulcers, spontaneously occurring peptic ulcers, and secondary prevention settings. The AGA Clinical Practice Update generally does not recommend the routine use of P-CABs as first-line therapy for the treatment or prophylaxis of peptic ulcer disease [18]. However, given their rapid and potent acid suppression, P-CABs may have potential advantages in specific clinical settings, particularly in the early phase of ulcer management or in high-risk situations, as discussed in the following sections.

#### Artificial ulcers

In post-endoscopic artificial ulcers, the primary clinically relevant outcome is delayed bleeding, which directly affects clinical management after endoscopic treatment, in addition to ulcer shrinkage and healing.

Several studies have shown that vonoprazan achieves ulcer shrinkage and complete healing rates that are comparable to those of PPIs, with some studies suggesting faster early healing or the potential to shorten the treatment duration. However, many of these studies are relatively small, and a meta-analysis has suggested that the overall incremental benefit of P-CABs over PPIs is modest [91–96].

In terms of delayed bleeding, the incidence appears to be largely comparable between P-CABs- and PPI-based regimens [95, 97–99]. Notably, Kato et al. demonstrated that a shortened 3-week course of vonoprazan did not increase delayed bleeding compared with the conventional 8-week treatment duration [7]. These findings suggest that intensive acid suppression in the early phase after endoscopic treatment may be more critical than prolonged therapy, with potential clinical and economic advantages.

Overall, P-CABs appear to be as effective as PPIs for post-endoscopic ulcer management; however, their superiority in reducing delayed bleeding remains to be fully established.

#### Spontaneously occurring peptic ulcers

Several randomized controlled trials have evaluated P-CAB-based therapies for spontaneously occurring peptic ulcers. Overall, vonoprazan demonstrated non-inferior efficacy to PPIs in terms of endoscopic healing rates for both gastric and duodenal ulcers [100–102].

Similar findings have been reported for other P-CABs, such as tegoprazan and zastaprazan, which have healing rates that are comparable to those of PPI-based therapies [103, 104].

Current evidence suggests that P-CABs provide at least equivalent efficacy to PPIs in the treatment of peptic ulcers; however, clear superiority has not been consistently demonstrated.

#### Secondary prevention

In the context of secondary prevention, several randomized controlled trials have evaluated P-CABs for the prevention of ulcer recurrence in patients receiving low-dose aspirin (LDA) or non-steroidal anti-inflammatory drugs (NSAIDs) [105, 106].

In patients receiving LDA, vonoprazan demonstrated non-inferior efficacy compared with PPIs. In a phase III trial, the 24-week ulcer recurrence rate was 2.8% with lansoprazole compared with 0.5% and 1.5% with 10 and 20 mg vonoprazan, respectively [105].

Similar findings have been reported in patients receiving NSAIDs. In a phase III trial, the incidence of endoscopically confirmed ulcers at 24 weeks was 5.5% with lansoprazole compared with 0.5% and 1.5% with 10 and 20 mg vonoprazan, respectively [106]. Comparable findings have also been reported with fexuprazan, with ulcer incidence rates of 3.4% for fexuprazan compared with 3.0% for lansoprazole at 24 weeks [107].

A randomized trial from Thailand comparing vonoprazan with intravenous PPI therapy also reported no significant differences in ulcer prevention outcomes [108].

These findings suggest a consistent trend toward lower ulcer recurrence rates with vonoprazan than with PPIs; however, the superiority of vonoprazan has not yet been formally established. Overall, P-CABs represent an effective alternative to PPIs for the secondary prevention of both LDA- and NSAID-associated ulcer risk.

### Other indications

#### Functional dyspepsia (FD)

FD is characterized by chronic upper gastrointestinal symptoms without identifiable structural abnormalities. Its pathophysiology is multifactorial and includes impaired gastric accommodation, delayed gastric emptying, and visceral hypersensitivity [109–115]. Therefore, symptom improvement is the primary therapeutic goal and outcome measure.

According to current guidelines, including the Rome criteria and related consensus statements, PPIs are recommended as first-line therapy for FD, particularly for both epigastric pain syndrome and post-prandial distress syndrome [109, 116]. Given the more potent and sustained acid suppression achieved by P-CABs, an important clinical question is whether P-CABs could provide superior symptom control compared with PPIs in patients with FD.

Although several prospective studies have suggested that P-CABs, such as vonoprazan and tegoprazan, may improve the symptoms of FD, direct randomized comparisons with PPIs are lacking [117–121]. Therefore, there is currently insufficient evidence to support the preferential use of P-CABs over PPIs in patients with FD, and further comparative studies are required.

#### Eosinophilic esophagitis (EoE)

EoE is a chronic immune-mediated esophageal disease characterized by esophageal dysfunction and eosinophilic infiltration. PPIs are currently recommended as the first-line therapy [122, 123].

The primary treatment goals are improvement of symptoms, particularly dysphagia, and histological remission. Studies suggest that vonoprazan may be effective in achieving these outcomes [124, 125]. However, the available evidence is limited, and high-quality randomized controlled trials comparing P-CABs with PPIs are lacking.

In addition, differences in disease phenotype and pathophysiology between Asian and Western populations have been reported, which may influence the treatment response [126]. Therefore, there is currently no clear evidence to support the preferential use of P-CABs over PPIs for the treatment of EoE, and further studies are warranted.

## Potential risks associated with the long-term use of P-CABs

To date, clinical trials have consistently demonstrated that the short-term use of P-CABs is generally well tolerated, with adverse event rates comparable to those of PPIs [127]. However, these findings are largely based on studies with relatively short observation periods, and data on long-term safety remain limited.

PPIs have been associated with various potential adverse effects, including an increased risk of infections (e.g., *Clostridioides difficile* infection and pneumonia), fractures, renal impairment, and nutritional deficiencies (e.g., magnesium, vitamin B_12_, and iron) [128] [129–131]. Gastrointestinal changes such as fundic gland polyps likely related to hypergastrinemia and concomitant enterochromaffin-like (ECL) cell hyperplasia have also been reported [132–134]. Associations with dementia, mortality, metabolic disorders, and small intestinal bacterial overgrowth have also been suggested; however, causal relationships remain controversial [135–138].

Given their more potent and sustained acid suppression, there is concern that the long-term use of P-CABs may confer similar or potentially greater risks than PPIs. Follow-up studies on vonoprazan have not demonstrated a clear increase in gastric cancer incidence or ECL cell hyperplasia progression over mid-term observation periods of up to 5 years; nevertheless, consistently elevated serum gastrin levels have been observed [139–141]. Importantly, these data are largely derived from studies in *H. pylori*-negative populations. Recent observational cohort studies have suggested a potential association between vonoprazan use after *H. pylori* eradication and an increased risk of gastric cancer; however, these findings should be interpreted with caution because of inherent confounding and indication bias [142, 143]. Notably, a large population-based cohort study also reported that long-term PPI use was associated with an increased risk of gastric cancer even after *H. pylori* eradication (hazard ratio, 2.44), suggesting that this association may not be unique to vonoprazan, but could be related to potent acid suppression [144]. Given that *H. pylori* infection combined with long-term acid suppression may promote the progression of corpus atrophy, a test-and-treat strategy should be considered before prolonged therapy [145].

By contrast, long-term safety data for other P-CABs remain limited, given that most of the available studies are based on relatively short-term clinical trials. Hypergastrinemia is a consistent pharmacodynamic effect of P-CAB therapy and appears to be reversible after treatment discontinuation [146]. However, its broader clinical significance remains unclear. In particular, its independent effects on gastrointestinal motility, endocrine function, and systemic diseases have not been clearly established, and many reported associations are likely confounded by concomitant hypochlorhydria or underlying conditions [147].

Although the short-term safety profiles of P-CABs appear comparable to those of PPIs, the long-term health implications of sustained and potent acid suppression, particularly in relation to hypergastrinemia, remain unclear. Therefore, the cautious use of long-term P-CAB therapy is warranted, particularly in patients requiring prolonged treatment.

## Future perspectives and unresolved clinical questions

Despite significant advances in acid-suppressive therapy, several important clinical questions remain unanswered. In particular, whether increasingly potent acid suppression directly translates to meaningful clinical benefits in all patient populations remains unclear. As the use of P-CABs increases, it is essential to reconsider the fundamental assumptions underlying acid-suppressive treatment strategies and critically evaluate their appropriate role in clinical practice.

**Fig. 1:**
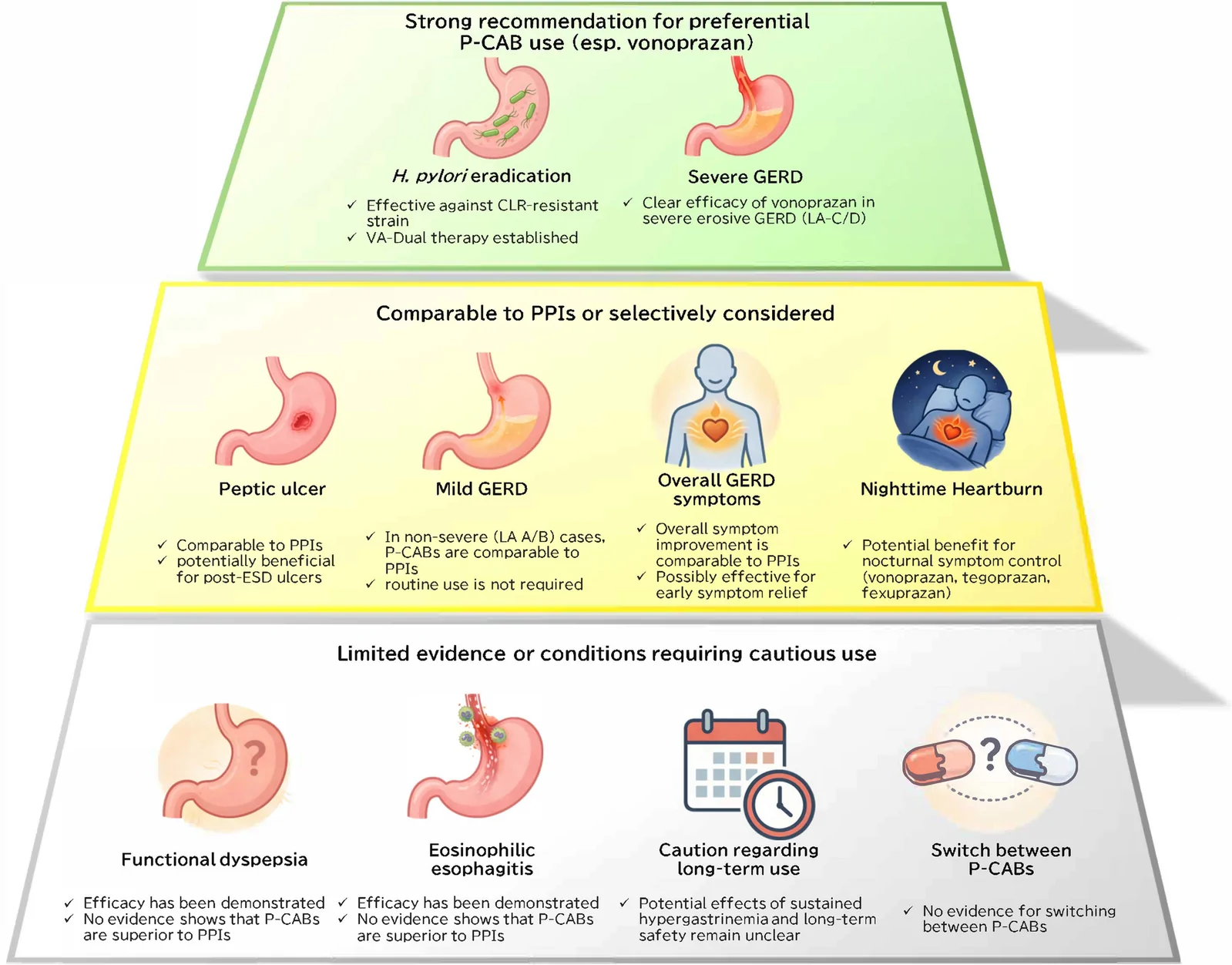
Clinical positioning of P-CABs across acid-related diseases. This schematic summarizes the current evidence regarding the clinical positioning of P-CABs compared with PPIs across major acid-related disorders. P-CABs, particularly Vonoprazan, have demonstrated advantages in *H. pylori *eradication therapy and severe GERD (Los Angeles grade C/D), whereas their efficacy appears largely comparable to that of PPIs in non-severe GERD and peptic ulcer disease. Evidence remains limited for functional dyspepsia and eosinophilic esophagitis. *P-CAB* potassium-competitive acid blocker, *PPI* proton pump inhibitor, *GERD* gastroesophageal reflux disease, *LA* Los Angeles classification, *CLR* clarithromycin, *VA-dual therapy* vonoprazan-amoxicillin dual therapy, *ESD* endoscopic submucosal dissection

A key issue is the need to clearly define therapeutic goals. The clinical value of more potent acid suppression depends on whether the primary goal is symptom relief or mucosal healing. Notably, symptom improvement and histological or endoscopic healing are not always correlated, particularly in conditions such as GERD or EoE [123, 148]. In patients whose management is primarily symptom-driven, the incremental benefit of stronger acid suppression may be limited, thus raising questions about the necessity of the routine use of more potent agents in such settings (Fig. 1).

Another important gap in the evidence concerns the comparative efficacy of different P-CABs. To date, direct head-to-head studies are limited. Outside this context, robust comparative data remain scarce, thus making it difficult to determine whether clinically meaningful differences exist between these agents. Furthermore, the clinical significance of switching between P-CABs has not been established, and it remains unclear whether such strategies provide additional benefits beyond the initial therapy.

Long-term safety also remains an important consideration. Although P-CABs are generally well tolerated in the short term, the clinical implications of sustained hypergastrinemia and prolonged potent acid suppression remain to be fully elucidated. Long-term studies are required to better define their safety profile. In addition, an experimental study suggested that profound acid suppression may influence the gastric mucosal barrier, including potential alterations in the mucus layer; however, its clinical relevance remains unclear, although such changes may potentially be related to the pathophysiology of mucosal hypersensitivity [149].

Therefore, more potent acid suppression is not necessarily synonymous with better clinical outcomes. Rather, the optimal use of acid-suppressive therapy should be guided by clearly defined therapeutic goals that balance efficacy and safety. In addition to efficacy and safety, cost considerations may also influence therapeutic decision-making in real-world practice, particularly in long-term maintenance settings. Drug pricing varies substantially across regions; for example, a recent cost-effectiveness analysis in the United States demonstrated that P-CAB-based therapy was associated with an additional cost of approximately $3,240 compared with PPIs in GERD management [18, 150]. This disparity suggests that cost–benefit considerations may be particularly important in certain healthcare systems. In this context, an individualized, indication-based approach is essential to avoid the unnecessary escalation of therapy while maximizing clinical benefits. Further studies are required to clarify the long-term safety and appropriate positioning of P-CABs in clinical practice.

## Conclusion

P-CABs should be preferentially considered in clinical settings where their superiority has been clearly demonstrated, particularly for short-term therapy such as *H. pylori* eradication and in patients with severe erosive GERD, where vonoprazan has shown robust efficacy. However, their routine use for long-term management requires caution.

Outside of these settings, evidence for P-CABs in GERD remains inconsistent despite potential benefits, such as improved nocturnal symptom control; furthermore, their superiority over PPIs has not been consistently established. In peptic ulcer disease, P-CABs provide healing rates that are broadly comparable to those of PPIs, although potential advantages in specific settings, such as early phase management and post-endoscopic bleeding, have been suggested. In FD and EoE, current evidence remains limited and does not support the preferential use of P-CABs over PPIs.

Accordingly, escalation to a more potent acid suppression should not be routinely adopted, particularly in symptom-driven conditions. Treatment strategies should also be individualized. On-demand therapy may be appropriate for patients who achieve adequate symptom control.
